# A Possible Effect of Concentrated Oolong Tea Causing Transient Ischemic Attack-Like Symptoms

**DOI:** 10.9734/BJMMR/2013/4703

**Published:** 2013-07-18

**Authors:** John W. Layher, Jon S. Poling, Mayumi Ishihara, Parastoo Azadi, Gerardo Alvarez-Manilla, David Puett

**Affiliations:** 1 Oconee Heart and Vascular Center, St. Mary’s Health Care System, Inc., Athens, GA 30606, USA; 2 Athens Neurological Associates, Athens, GA 30606, USA; 3 Division of Analytical Services, Complex Carbohydrate Research Center, University of Georgia, Athens, GA 30602, USA; 4 Bioanalytical Lab, Pharmaceutical Product Development, Richmond, VA 23230, USA; 5 Department of Biochemistry and Molecular Biology, University of Georgia, Athens, GA 30602, USA; 6 Department of Biochemistry and Biophysics, University of North Carolina at Chapel Hill School of Medicine, Chapel Hill, NC 27599, USA

**Keywords:** Transient ischemic attack, oolong tea, mass spectrometry, high performance liquid chromatography

## Abstract

**Aims:**

Tea (green, oolong, and black) is the second most widely consumed beverage worldwide, second only to water. Aside from a few reported adverse effects, tea, particularly green tea, appears to be beneficial for human health. In the case described herein, a male experienced several transient ischemic attack-like symptoms immediately following the consumption of a cup of high quality oolong tea. A thorough medical evaluation uncovered no evidence of such an attack and leads to the suggestion of a heretofore unreported response to oolong tea.

**Presentation of Case:**

A 72-year old male with hypertension and atrial fibrillation, who takes valsartan/hydrochlorothiazide to control hypertension and warfarin to reduce the risk of thrombosis and thromboembolism, presented at the emergency room of a local hospital describing several transient ischemic attack-like symptoms immediately after consuming a cup of oolong tea. His symptoms included presyncope, disequilibrium, bilateral hand parathesias, mild dysphasia, and visual problems (but apparently not presbyopia or amaurosis fugax), all of which had disappeared in approximately two hours after drinking the tea. (Mild presyncope was previously noted by the patient when ingesting a strong green tea.) No unusual features emerged from his physical examination, and his blood work was unremarkable except for elevation of his partial thromboplastin time (39 sec) and prothrombin time (22.5 sec), giving an international reference of 2.0, all consistent with the effects of warfarin. A battery of tests by the emergency room physician, a cardiologist, and a neurologist, e.g. electrocardiogram, brain computerized tomography, 2-dimensional transthoracic echocardiogram, brain magnetic resonance imaging, with and without 20 ml Gadolinium, and a magnetic resonance angiogram, confirmed the earlier diagnosis of atrial fibrillation but disclosed no additional malfunction in his heart. His brain showed no evidence of a prior hemorrhage, and his carotid arteries were clear.

**Methodology and Results:**

Analysis of the oolong tea by high performance liquid chromatography and mass spectrometry identified the major catechins and two methylxanthines, caffeine and theophylline, as well as other constituents, but there was no evidence of any extraneous chemicals that could lead to the symptoms.

**Conclusion:**

In view of the rapid onset of symptoms after the consumption of oolong tea, bilateral as opposed to unilateral parathesis, and the absence of any evidence of a hemorrhage or the presence of impurities in the tea, we suggest that the transient ischemic attack-like symptoms could possibly be attributable to one or more components of the oolong tea and was not an atypical magnetic resonance imaging-negative transient ischemic attack.

## 1. INTRODUCTION

Tea, one of the most widely consumed beverages worldwide, is derived from young buds and the leaves of several varieties of *Camellia sinensis* (Theaceae), first cultivated in China and Southeast Asia over 2,000 years ago [1]. The three major types of tea are green, oolong, and black. Oolong tea, upon which this paper is based, represents an intermediate in the preparation of green and black teas. Green tea is prepared by initial heating (pan firing or steaming) to inactivate the endogenous enzymes, while oolong and black teas are said to be ‘fermented,’ that is, after moisture reduction (a process termed withering), the leaves are rolled and crushed, a procedure that begins the endogenous enzymatic reactions. Oolong tea is fired soon after crushing to inactivate these reactions, while black tea is prepared after increased ‘fermentation.’ Oolong tea, like the green and black teas, is composed primarily of the four polyphenolic catechins, epigallocatechin gallate (EGCG), epigallate catechin, epicatechin gallate, and epicatechin, with EGCG being the most prevalent. While there are many studies on green tea, the literature on oolong tea is less detailed. There is an indication that oolong tea may contain several unique components resulting from the fermentation protocol, i.e. oolongtheanin, 8-ascorbyl EGCG, and oolonghomobisflavans [1], but no suggestions of physiological effects have been attributed to these products.

In addition to its popularity as a beverage, tea has been reported to have many beneficial effects to human health, including a reduction in the incidence of cancer and cardiovascular disease [2–7] as well as suggestions that tea may reduce the possibility of ischemic stroke [8–11]. Further, it has been shown that oolong tea is more effective than black tea, although less effective than green tea, in inhibiting an important enzyme in blood pressure regulation, angiotensin converting enzyme [12]. These reports notwithstanding, caution has been suggested in recommending tea to the population at large or to individual patients to lower cardiovascular risks [13]. Although there are some indications of adverse effects from concentrated green tea extracts, including liver toxicity and interactions with drugs and herbal supplements [14], there are no reported symptomatic responses to oolong, green, or black tea similar to those detailed in the present case study.

There are many neurologic symptoms accompanying a transient ischemic attack (TIA), and the etiology can be diverse, including hypertension, atrial fibrillation, hypercholesterolimia, diabetes, increasing age, and a family history of stroke. A TIA arises from ischemia, most often from an embolus originating in an atherosclerotic plaque in one of the carotid arteries or from a thrombus in someone with atrial fibrillation. Indicative of a possible future stroke, preventive action should be taken to minimize such an occurrence. Since atrial fibrillation is one of the factors responsible for a TIA, an anticoagulant such as warfarin or one of the newer anti-coagulants is often prescribed for such patients. Warfarin exerts its action on the vitamin K-dependent clotting factors, in particular by inhibiting the essential enzyme, vitamin K epoxide reductase complex 1 (VKORC1), that acts in the vitamin K cycle to catalyze the reduction of vitamin K epoxide to vitamin K quinone, thus diminishing the carboxylation of factors II, VII, IX, X, and the C and S anticoagulant proteins [15,16].

The case presented in this study is suggestive of a possible effect of concentrated oolong tea producing some TIA-like symptoms that, to the best of our knowledge, have not been reported before.

## 2. PRESENTATION OF CASE

### 2.1 Evaluation by an Emergency Care Physician

The patient, a 72 year-old Caucasian male in good physical condition, presented at the emergency department of a local hospital describing neurologic symptoms that had occurred about two hours earlier and were indicative of a TIA. He reported that while speaking with someone in his office as he finished drinking a cup of oolong tea he developed a number of worrisome symptoms. The patient had consumed a cup of green or oolong tea daily for many years prior to this incident, the usual amount being approximately 250 mg (dry weight of tea leaves) per 240 mL hot water. On this particular day the tea was prepared in a more concentrated blend, about 4–5 times the usual (1–1.2 g dry weight per 240 mL), seeped five minutes, the leaves remaining in the cup during the 30 min period of consumption. Immediately upon finishing the cup of tea, he experienced the following indicators of a TIA: mild visual problems (symptoms not descriptive, however, of presbyopia or amaurosis fugax), presyncope, disequilibrium, hand parathesias (albeit bilateral), and following that meeting noticed some mild dysphasia when speaking with someone else. He also noted that he had experienced some presyncope in the past when consuming either a greater quantity or a more concentrated Japanese green tea at a local restaurant. The symptoms had disappeared upon presentation at the hospital, but there was sufficient concern on the part of the patient, his wife, and attending physician that they felt an evaluation was needed in view of his past medical history. He had been diagnosed with hypertension and benign prostatic hypertrophy, and five years earlier with atrial flutter. The flutter was corrected by cardiac ablation in the right atrium, with atrial fibrillation one year earlier. His daily medications were warfarin sodium (coumadin, 5 mg six days a week and 7.5 mg once a week), taken in the evening about 9:00 PM, and valsartan/hydrochlorothiazide (diovan/HCTZ, 160 mg/12.5 mg), alfuzosin HCI (uroxatral, 10 mg), rebeprazole sodium (aciphex, 20 mg for acid reflux), and bifidobacterium infantis (probiotic, 4 mg), all taken in the early morning between 5:00–6:00 AM. He was alert and had a temperature of 97.3°F, a pulse rate of 57 beats per minute, a systolic/diastolic blood pressure of 160/90 that later decreased to 139–141/80–76 mm Hg during his stay in the emergency department, and an oxygen saturation of 97–99% throughout his stay as an outpatient. He mentioned that his blood pressure had been treated for several years and was generally lower than that measured in the hospital setting. Laboratory tests were mainly in the normal range, with a few being either marginally low or high. The prothrombin and partial thromboplastin times were elevated (22.5 and 39 sec, respectively), and the prothrombin time-international reference (PT-INR) was 2.0, attributable to warfarin. An x-ray of the chest demonstrated that the lungs were clear bilaterally and the cardiac silhouette and mediastinum were unremarkable; CT of the brain (axial imaging) showed no evidence of an acute process. Heart irregularity was noted, and an electrocardiogram (EKG) revealed atrial fibrillation with an average rate in the 50s, a normal axis, and no evidence of ST segment abnormalities. The patient was administered IV fluid and then released with a recommendation that he see a cardiologist for further evaluation.

### 2.2 Evaluation by a Cardiologist

A physical examination by the cardiologist demonstrated an irregularly irregular rhythm, consistent with the patient’s known atrial fibrillation, but no abnormalities. The carotids were full and equal bilaterally without bruits; the neck veins were normal with no elevation of the jugular venous pressure. The conclusions from a 2D transthoracic echocardiogram (TTE) using color and doppler flow imaging were as follows: there was mild right atrial enlargement with mild to moderate tricuspid insufficiency; the estimated right ventricular systolic pressure was slightly elevated; the left and right ventricles appeared normal in size, and the left ventricular systolic and diastolic function appeared normal; the aortic valve showed mild sclerosis without evidence of stenosis; and the mitral valve was structurally normal. An agitated saline contrast image (‘bubble study’) showed no evidence of an intracardiac shunt. It was recommended that the patient begin 81 mg aspirin to be taken 3 times a week, along with warfarin. Overall there was no evidence of serious cardiac problems other than atrial fibrillation. In view of the TIA-like symptoms the patient originally presented with, it was suggested that he also be examined by a neurologist.

### 2.3 Evaluation by a Neurologist

A brain magnetic resonance imaging (MRI), with and without 20 ml Gadolinium, and a magnetic resonance angiogram (MRA) yielded the following information. The diffusion-weighted images were negative, and gradient echo studies showed no evidence for a prior hemorrhage. Overall, no acute intracranial abnormality was observed, and, importantly, there was no evidence for a prior cortically-based infarct; moreover, after Gadolinium administration a normal meningeal and vascular enhancement was observed. There was evidence of minimal small vessel ischemic cerebrovascular changes in the hemispheres, some slight fluid accumulation within the mastoid air cells, and mucosal lining thickening in the inferior maxillary sinuses and a few of the ethmoidal air cells. The MRA documented the absence of any hemodynamically significant stenosis involving the arteries of the neck.

### 2.4 Summary

A brain CT and MRI failed to detect any evidence of an infarct, and a MRA of the neck indicated normal flow. The 2D TTE of the heart, with and without agitated saline to ascertain bubble passage, detected no abnormality. Thus, atrial fibrillation appeared to be the only significant problem of the patient.

Based on these medical results, a study was undertaken to characterize the oolong tea that was consumed and, although there is no known precedent in the literature, could possibly have led to the acute TIA-like symptoms experienced by the patient. The goal was to determine the constituents in the tea, after extraction in hot water as was done for consumption. The techniques employed were liquid chromatography tandem mass spectrometry (LC-MS/MS) and high performance liquid chromatography (HPLC) using conditions similar to those described by others [17–20]. The methodologies used and the experimental data are reported in the Appendix.

## 3. RESULTS AND DISCUSSION

**Fig. 1:**
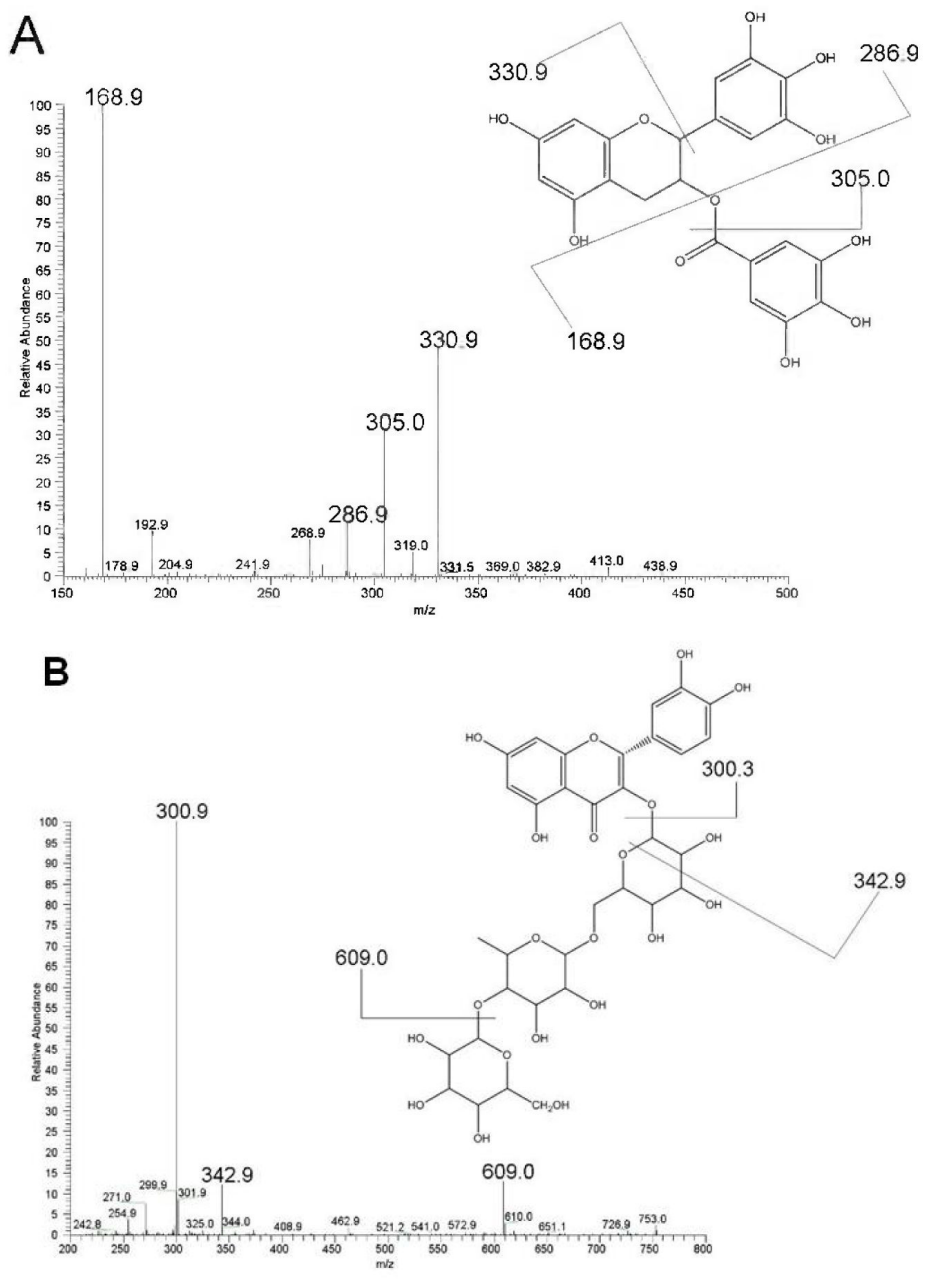
MS/MS spectra of oolong tea in the negative ion mode documenting the presence of EGCG, m/z 457 (A), and quercetin 3-O-glucosylrutinoside, m/z 771 (B). The main fragment ions identified in panel A are gallic acid (m/z 168.9) and epigallocatechin (m/z/305); the neutral loss of trihydroxybenzene is also indicated (m/z 331). In panel B the main fragment ion identified is quercetin (m/z 301). A neutral loss of the carbohydrate portion is indicated at m/z 349, arising from a cross-ring cleavage of quercetin at the 1 and 3 carbon bonds, and at m/z 609, attributed to a neutral loss of the terminal hexose

Selected MS/MS spectra of the oolong tea extract are presented as examples in Appendix Fig. 1. Fig. 1A shows the MS/MS spectrum of EGCG analyzed in negative ion mode at m/z 457 (eluted at 12.55 min). The mass-to-charge ratios (m/z) of the main fragment ions observed were 168.9 and m/z 305, corresponding to gallic acid and epigallocatechin, respectively. Also, EGCG showed a neutral loss of trihydroxybenzene at m/z 331. The MS/MS spectrum of quercetin 3-O-glucosylrutinoside at m/z 771 (eluted at 16.87 min) is given in Fig. 1B. The main fragment ion (m/z 301) corresponds to quercetin, confirming the structure of the flavonoid moiety in the parent ion. A neutral loss of the carbohydrate portion was found at m/z 349 and m/z 609. The m/z 349 component is attributed to a cross-ring cleavage of the hexose attached on quercetin at the 1 and 3 carbon bonds (1,3A/1,3X), whereas m/z 609 results from a neutral loss of the terminal hexose. This fragmentation pattern confirmed the structure of the carbohydrate moiety in the parent ion. A few flavonoids, including caffeine, were detected in just the positive ion mode because of their polarity. Also, in the positive ion mode acylated flavonol glycosides were observed in the H^+^, Na^+^, and K^+^ forms. For MS/MS analysis of the acylated flavonol glycosides in the positive ion mode, the H^+^ form caused ion suppression during MS/MS fragmentation that resulted in poor signals; in these cases structural assignments were based on the Na^+^ adducts. The flavonoids identified in the tea sample are given in Appendix Tables 1 and 2 for results obtained in the negative and positive ion modes, respectively. For structural assignment the observed full mass and MS/MS were compared to those of known standards, those reported previously [18–20], and the theoretical full mass and MS/MS.

A sample of Yamamotoyama oolong tea was brewed according to the recommendations of the manufacturer, filtered, and then analyzed by MS. This control tea gave an almost identical profile to that of the oolong tea consumed by the patient, confirming that unusual or unexpected peaks were not present in the ‘experimental’ tea.

**Fig. 2:**
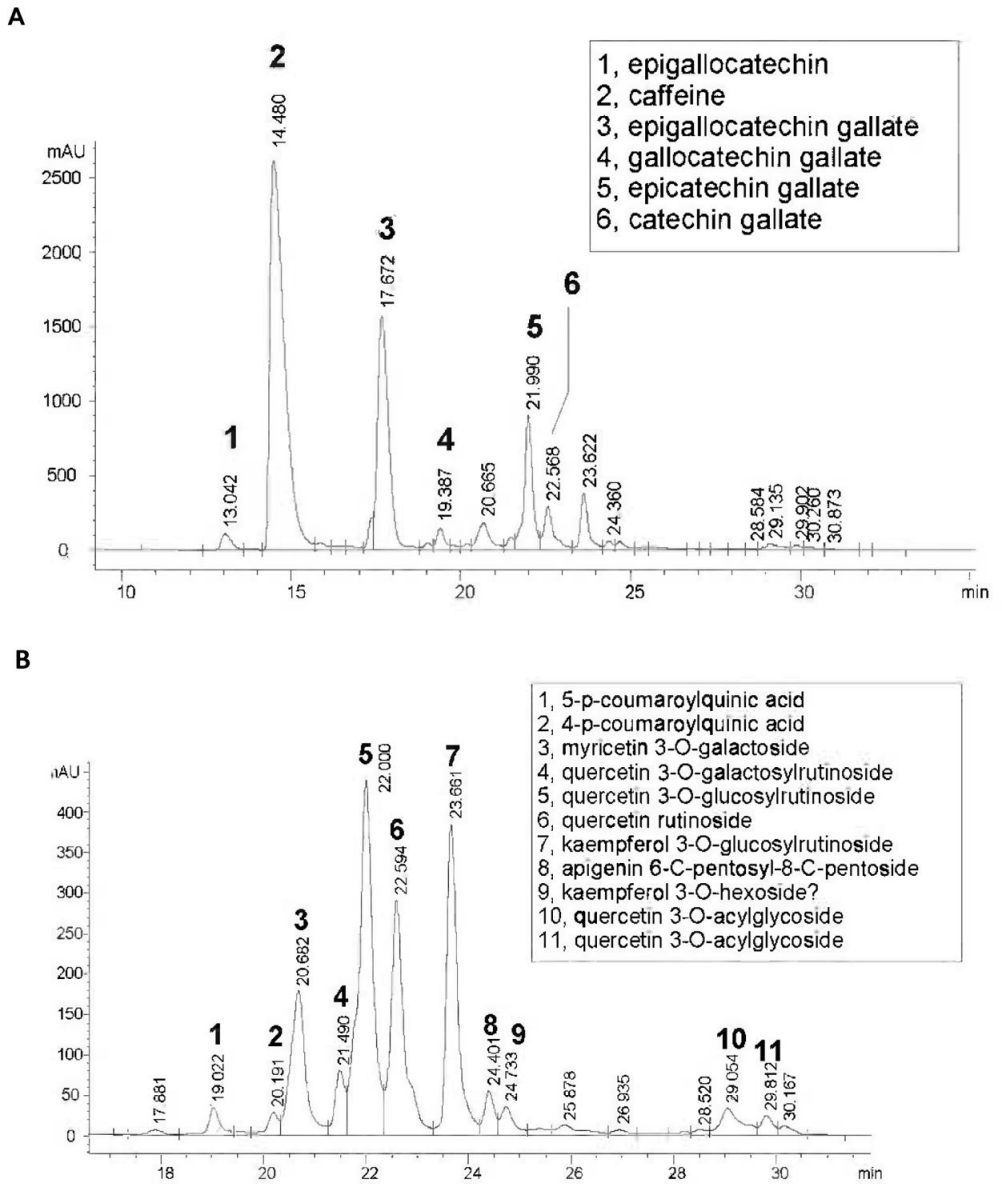
HPLC chromatograms of oolong tea monitored at 270 nm (A) and 350 nm (B). See Methods for experimental details, and the major peaks are identified

The HPLC chromatograms of the oolong tea extract are shown in Fig. 2 with detection at 270 nm (panel A) and 350 nm (Panel B), and assignments of the major peaks. Caffeine and the catchenins, EGCG and epicatechin gallate, are the major components identified at 270 nm, and the major phenolic components identified at 350 nm are myricetin 3-O-galactoside, quercetin 3-O-glucosylrutinoside, quercetin rutinoside, and kaempferol 3-O-glucosylrutinoside; many other minor components are seen in each chromatogram.

The results obtained from MS and HPLC characterization of the oolong tea are in excellent agreement with findings reported by others [18–20], demonstrating the complexity and variety of components present in tea. A detailed study on 41 green teas and 25 fermented teas enabled Lin et al. to classify the teas into five categories based upon their compounds [18]. Oolong tea was in Group 3 that, compared to green tea, contained less EGCG, more theaflavins and other catechins, and comparable amounts of glycosylated flavonols. The majority of the health benefits attributed to tea are believed to arise from the flavonoids, particularly the catechins (polyphenols) that are the major constituent of tea. Present in much lower concentrations are the methylxanthines, including caffeine, theophylline, and methlyxanthine theobromine, the first two of which were detected in our sample. While caffeine, a well-known CNS and metabolic stimulant, may be suspect in leading to some of the TIA-like symptoms experienced by the patient, there is no documentation of such effects in the literature. Four caffeine-induced disorders are recognized by the American Psychiatric Association, none of which correspond to the symptomology of the patient discussed herein [21]. Interestingly, EGCG, the major catechin in tea and confirmed to be present in the tea in question in this study, has been suggested to counteract caffeine-mediated stimulant effects [22].

Although tea leaves contain 14.3 and 2.6 μg vitamin K/g dry leaves in green and black tea, respectively [23], relatively little is extracted by seepage in hot water [24]. There are, however, two reports implicating tea, and thus vitamin K, in patients taking warfarin. In one, a male began consuming a rather large quantity of green tea daily, 0.5–1.0 gallons, and experienced a reduction in his INR from 3.8 to 1.4 that was restored to 2.6 upon discontinuation of green tea [25]. In another, a woman had regularly drunk an undisclosed amount of black tea each day and maintained her INR between 2–3. Upon discontinuing consumption of black tea, her INR increased to 5.0 but was restored to the 2–3 range when her warfarin dose was decreased [26]. While suggesting that tea can alter the INR of some individuals taking warfarin, it was not an issue in the present case since the patient was found to have an INR of 2.0 when he presented at the hospital.

It is unlikely that the response to the tea could arise from an acute metabolic disturbance of one or more medicines being taken by the patient, or vice versa, particularly since the onset of the symptoms appeared immediately after drinking the tea while the medications had been taken anywhere from 7–15 hours earlier. There are reports in experimental animals that quercetin enhances the bioavailability of valsartan [27]; however, the patient described herein had taken his daily valsartan some seven hours before drinking the concentrated tea. In another report based on studies in human hepatoma HepG2 cells and primary cultures of human hepatocytes, the authors concluded that EGCG is an antagonist to the aryl hydrocarbon receptor that regulates the expression of the cytochrome (CYP) P450 subfamily CYP1A, composed of two members, CYP1A1 and CYP1A2, that metabolize many chemical carcinogens [28]. At present, there is no apparent correlation of this effect with any of the symptomology experienced by the patient. Additionally, genotypes of CYP2C8 and CYP2C9, cytochrome P450s responsible for the metabolism of most angiotensin II receptor antagonists, impact on the pharmacokinetics of valsartan [29], but the relationship of these findings to the patient presented is not known. Of interest are the reports indicating that tea may reduce the onset of stroke [8–11], thus strengthening our suggestion that the symptoms observed in the patient were, in many respects, TIA-like, not an actual TIA.

Since (a) atrial fibrillation significantly increases a person’s risk of stroke or systemic embolization, with the risk steadily increasing with age, and (b) while warfarin reduces the risk some 62–68%, but does not eliminate it altogether, atrial fibrillation must be considered as a possible cause of a TIA in the patient. Moreover, brain and MR angiography imaging have known limitations, including (a) the ability to visualize some vessels but not others, and (b) the possibility that some microemboli could have been present but were too small to be imaged or the emboli may have broken up and resolved by the time the imaging was performed. On the other hand, the neurologic symptoms exhibited by the patient do not fit any cerebral vascular distribution involving, for example, a clot going to one area of the brain. In light of the fact that some of the symptoms were bilateral, e.g. hand parathesias, while others suggest a global cerebral process, e.g. presyncope, only a simultaneous diminished flow or pressure going to both sides of the brain could cause a similar picture, and this is ruled out by the normal or slightly elevated blood pressure of the patient in the Emergency Department. Thus, following a thorough evaluation of the patient and a critical review of the medical findings, no unequivocal evidence was found to indicate that a TIA had occurred, and it must be considered that the symptomology could be attributable to an unusual acute effect from the concentrated oolong tea.

## 4. CONCLUSIONS

A thorough medical evaluation revealed no indications of a TIA, or even an arterial territory syndrome, and no unexpected components were identified in the oolong tea by HPLC and LC-MS/MS. We suggest that concentrated oolong tea may, under certain conditions, lead to acute TIA-like effects. Such a response to tea is unusual, and it is not known if the symptomology described could arise from just one or several constituents of the tea. Moreover, it is not known whether or not there is an interaction of the oolong tea with one or more of the medications taken by the patient or if an unusual pharmacogenetic background of the patient could enhance any possible effect of the tea. While one could argue that tea-mediated hypotension could explain some of the symptoms experienced by the patient, there is no conclusive evidence that a drop in blood pressure could explain all the symptoms. Thus the effect may reflect both hemodynamic and CNS effects attributable to the oolong tea.

## Figures and Tables

**Table 1:** Flavanoid analysis of oolong tea sample by LC-MS/MS in the negative ion mode

RT	m/z	Proposed structure	MS/MS
2.30	173	theamine	-
7.31	305	gallocatechin	179, 221, 261
12.3	337	3-p-coumaroylquinic acid	173, 191
12.41	289	catechin	245
12.44	305	epigallocatechin	287
12.55	457	epigallocatechin gallate	169, 331, 305
13.34	337	5-p-coumaroylquinic acid	173
14.34	593	6,8 C-glycosylapigenin	473, 353, 503, 383, 575
14.46	457	gallocatechin gallate	169, 331, 305
14.64	337	4-p-coumaroylquinic acid	191
15.13	787	myricetin 3-O-galactosylrutinoside	317 (M), 769, 625 (Loss of hex), 479 (Loss of hexdeoxyhex)
15.31	479	myricetin 3-O-galactoside	316, 317 (M)
15.50	625	myricetin 3-O-rhamnosylglucoside	316, 317 (M), 479 (Loss of deoxyhex)
15.57	479	myricetin 3-O-glucoside	316, 317 (M)
15.68	563	apigenin 6-C-pentosyl-8-C-hexoside	443, 473 (Loss of C-glycoside), 383, 353, 503, 545
16.35	771	quercetin 3-O-galactosylrutinoside	301 (Q), 609 (Loss of hex)
16.65	593	--	413, 293
16.87	771	quercetin 3-O-glucosylrutinoside	301 (Q), 609 (Loss of hex)
16.92	787	myricetin 3-O-galactosylrutinoside	317 (M), 769, 625 (Loss of hex), 479 (Loss of hexdeoxyhex)
17.13	609	quercetin-rhamnosylgalactoside	301 (Q), 489
17.36	463	quercetin 3-galactoside	301 (Q)
17.47	625	myricetin compound	317 (M), 607, 581
17.47	609	quercetin-rutinoside	301 (Q)
17.66	533	apigenin 6-C-pentosyl-8-C-pentoside	443, 473 (Loss of C-glycoside), 383, 353, 515
17.73	463	quercetin 3-glucoside	301 (Q)
18.36	593	kaempferol 3-hexosyldeoxyhexoside	285 (K), 255, 327 (hex2deoxyhex)
18.47	755	kaempferol 3-O-glucosylrutinoside	285 (K), 593 (Loss of hex)
18.59	771	quercetin 3-O-hexosylrutinoside	301 (Q), 609 (Loss of hex)
18.81	447	kaempferol 3-O-galactoside	284 (K), 327
19.05	533	apigenin 6-C-pentosyl-8-C-pentoside	443, 473 (Loss of C-glycoside), 383, 353, 515
19.29	609	quercetin 3-O-deoxyhexosylhexoside	301 (Q)
19.33	593	kaempferol 3-hexosyldeoxyhexoside	285 (K)
19.33	625	myricetin compound	317 (M), 299, 607
19.63	447	--a	403, 249, 267
20.18	417	kaempferol 3-O-pentoside	284, 285 (K), 327
24.08	1063	quercetin 3-O-acylglycoside	917 (Loss of coumaric acid), 771, 615, 447, 301 (Q)
24.47	917	quercetin 3-O-p-coumaroylhexosyldeoxyhexosylhexoside	771 (Loss of coumaric acid), 301 (Q)
24.49	1063	myricetin 3-O-acylglycoside	917 (Loss of deoxy-hex), 777, 317 (M)
24.63	1049	quercetin 3-O-acylglycoside	903 (Loss of coumaric acid), 301 (Q)
24.71	917	quercetin 3-O-p-coumaroylhexosyldeoxyhexosylhexoside	771 (Loss of coumaric acid), 301 (Q)
24.78	1033	quercetin 3-O-acylglycoside	887 (Loss of coumaric acid), 741, 747, 447, 301 (Q)
24.86	901	quercetin 3-O-p-coumaroylhexosyldeoxyhexosylhexoside	755 (Loss of coumaric acid), 609, 447, 301 (Q)
25.00	1063	myricetin 3-O-acylglycoside	917 (Loss of deoxy-hex), 741, 451, 317 (M)
25.19	1049	quercetin 3-O-acylglycoside	903 (Loss of coumaric acid), 301 (Q)
25.41	1033	coumaric acid compound	887 (Loss of coumaric acid), 747, 597, 451
25.59	1063	quercetin 3-O-acylglycoside	917 (Loss of deoxy-hex), 771, 615, 447, 356, 301 (Q)
25.71	901	kaempferol 3-O-p-coumaroyldideoxyhexosylhexoside	755 (Loss of coumaric acid), 615, 433, 285 (K)
25.78	917	quercetin 3-O-p-coumaroylhexosyldeoxyhexosylhexoside	771, 753 (Loss of coumaric acid), 301 (Q)
25.86	1033	--a	--
25.97	901	quercetin 3-O-p-coumaroylhexosyldeoxyhexosylhexoside	755 (Loss of coumaric acid), 615, 447, 301 (Q)
26.56	901	kaempferol 3-O-p-coumaroyldideoxyhexosylhexoside	755 (Loss of coumaric acid), 615, 285(K)

a Unknown.

Abbreviations: Hex, hexose; K, kaempferol; M, myricetin; Q, quercetin.

**Table 2:** Flavonoid analysis of oolong tea sample by LC-MS/MS in the positive ion mode

RT	m/z	Proposed structure	MS/MS
1.54	175	theamine	--
1.86	181	theophylline	137, 138
4.87	307	gallocatechin	139, 151, 289 (Loss of OH)
8.47	195	caffeine	138
11.98	291	catechin	123, 139
12.28	459	epigallocatechin gallate	139, 151, 289
13.04	339	4-p-coumaroylquinic acid	147
14.14	595	6,8 C-glycosylapigenin	457 (Loss of C-hexo-side)
14.30	459	gallocatechin gallate	139, 151, 289
15.23	789	myricetin 3-O-galactosylrutinosode	319 (M), 628, 482
15.42	481	myricetin 3-O-galactoside	319 (M)
15.57	627	myricetin 3-O-rhamnosylglucoside	319 (M), 481 (Loss of deoxyhex)
15.60	481	myricetin 3-O-glucoside	319 (M), 313, 153, 171
15.72	565	apigenin 6-C-pentosyl-8-C-hexoside	547, 529, 428 (Loss of C-hexoside)
16.31	773	quercetin 3-O-galactosylrutinoside	303 (Q), 611, 465
16.65	595	O-hexose+ C-hexosylapigenin	433 (Loss of hexose)
16.95	773	quercetin 3-O-glucosylrutinoside	303 (Q), 611, 465
17.10	611	quercetin-rhamnosylgalactoside	303 (Q), 465
17.14	579	O-deoxyhex+ C-hexosylapigenin	433 (Loss of deoxy-hex)
17.32	465	quercetin 3-galactoside	303 (Q)
17.44	611	quercetin-rutinoside	303 (Q), 465
17.52	579	O-deoxyhex+ C-hexosylapigenin	433 (Loss of deoxy-hex)
17.63	535	apigenin 6-C-pentosyl-8-C-pentoside	517, 499, 481, 469
17.70	465	quercetin 3-glucoside	303 (Q)
18.37	595	kaempferol 3-hexosyldeoxyhexoside	287 (K)
18.52	757	kaempferol 3-O-glucosylrutinoside	287 (K)
18.78	449	kaempferol 3-O-galactoside	287 (K)
19.04	535	apigenin 6-C-pentosyl-8-C-pentoside	517, 499, 481, 403, 385, 367
19.26	595	kaempferol 3-hexosyldeoxyhexoside	287 (K)
19.59	449	kaempferol 3-O-hexoside	287 (K)
20.04	419	kaempferol 3-O-pentoside	287 (K)
23.68	1065 (*1086*)	quercetin 3-O-acylglycoside	*941, 785* (Loss of Q), *639, 493, 331*
24.10	1065 (*1086*)	quercetin 3-O-acylglycoside	*942, 787* (Loss of Q), *771, 640, 475, 330*
24.25	1051 (*1073*)	quercetin 3-O-acylglycoside	*771* (Loss of Q), *639, 607, 475*
24.37	919 (*941*)	quercetin 3-O-p-coumaroylhexosyldeoxyhexosylhexoside	*639* (Loss of Q), *475*
24.48	903 (*925*)	kaempferol compound	*779, 477*
24.60	1065 (*1086*)	kaempferol 3-O-acylglycoside	*801* (Loss of K), *637, 621, 475, 391*
24.79	1051 (*1073*)	quercetin 3-O-acylglycoside	*771* (Loss of Q), *621, 607, 475*
25.06	1035 (*1057*)	kaempferol 3-O-acylglycoside	*771, 607, 475*
25.25	1065 (*1086*)	kaempferol 3-O-acylglycoside	*941, 801* (Loss of K), *639, 475, 331*
25.32	903 (*925*)	kaempferol 3-O-p-coumaroylhexosyldeoxyhexosylhexoside	*639* (Loss of K), *475*
25.36	919 (*941*)	quercetin 3-O-p-coumaroylhexosyldeoxyhexosylhexoside	*639* (Loss of Q), *475*

a Unknown.

Abbreviations: Hex, hexose; K, kaempferol; M, myricetin; Q, quercetin; RT, retention time. MS/MS analysis of acyl glycosides (italicized) were performed on sodium adducts, and the m/z values are italicized in parentheses.

## ABBREVIATIONS

CNS: central nervous system
CT: computerized tomography
CYP: cytochrome P450
EGCG: epigallocatechin gallate
EKG: electrocardiogram
HPLC: high performance liquid chromatography
INR: international normalized ratio (see PT-INR)
LC-MS/MS: liquid chromatography-tandem mass spectrometry
LTQ: linear trap quadropole
MS: mass spectrometry
MS/MS: tandem mass spectrometry
MRA: magnetic resonance angiogram
MRI: magnetic resonance imaging
m/z: mass-to-charge ratio
PT-INR: prothrombin time-international normalized ratio
TFA: trifluoroacetic acid
TIA: transient ischemic attack
TTE: transthoracic echocardiogram
VKORC1: vitamin K epoxide reductase complex 1

## APPENDIX. METHDOLOGY OF TEA ANALYSIS

### Preparation of the tea for LC-MS/MS and HPLC

The oolong tea leaves (about 1.2 g of Tie Guan Yin Oolong Tea, Iron Goddess of Mercy, a high grade oolong tea) were seeped in 240 mL of hot water under conditions as close as possible to those used in the tea that was consumed (see Section 2.1). The leaves and any particulates were then separated from the liquid in the cup by filtration, and the filtrate was stored overnight at 5°C.

A SepPak cartridge (1 g) was inserted into a vacuum manifold and fitted with a 50 mL plastic syringe and then activated with 10 mL methanol (MeOH) and equilibrated with 20 mL 0.1% (trifluoroacetic acid (TFA). The tea extract (50 mL) was added, and the unbound components were eluted with 200 mL 0.1% TFA. Following transfer of the cartridge to a collection manifold, the non-polar compounds were eluted with 3 mL MeOH and then dried in a Speed-Vac. Next the sample was dissolved in 1 mL MeOH, dried, and reconstituted in a mixture of 0.9 mL 0.1% formic acid and 0.1 mL acetonitrile (CH_3_CN). Following heating at 50°C for about 5 min to ensure a homogeneous liquid phase, the sample was diluted 10-fold in 10% CH_3_CN in 0.1% formic acid.

### LC-MS/MS

Several MS runs were made using MALDI-TOF-MS (Bruker Autoflex) and LC-MS (Perkin-Elmer SC/EX), after which it was decided to use LC-MS/MS on a LTQ mass spectrometer (ThermoFisher) equipped with an electrospray ion source. Using conditions similar to those described for separating polyphenols [17], the sample was diluted 5-fold with mobile phase A (0.2% acetic acid in water) and filtered with 0.2 μm filters (Nanosep, PALL). 5 μL of the sample was injected into a C18 column (2.1 × 100 mm, Grace Vydac) followed by separation using a 60 min gradient of increasing mobile phase B (80% CH_3_CN and 0.2% acetic acid in water) at a flow rate of 200 μL/min.

The instrument was tuned with EGCG and caffeine for negative ion mode and positive ion mode, respectively. The LTQ was run in the automatic mode collecting a MS scan (full mass spectrometry at 150–2,000 m/z) followed by data-dependent MS/MS scans of the six highest abundant precursor ions. The data were analyzed manually for structural assignment, and the observed full mass and MS/MS were compared to those of known standards, e.g. EGCG and caffeine, those reported by others [18–20], and the theoretical full mass and MS/MS.

### HPLC

Analysis by HPLC was conducted to confirm the assignments made from LC-MS/MS and to check for the presence of any additional major peaks. The sample (10 μL) was injected into the same column used for LC-MS/MS and developed similarly. UV detection (Agilent Technologies) was at 270 nm and 370 nm, encompassing two separate runs. Using EGCG and caffeine as standards and the knowledge of components from LC-MS/MS, reports by others [18–20], and technical notes available from HPLC/MS companies, e.g. Agilent and Shimadsu, structural assignments were made.
